# 0353. Do the noble gases helium and argon exert neuroprotective effects in a rodent cardiac arrest model?

**DOI:** 10.1186/2197-425X-2-S1-P19

**Published:** 2014-09-26

**Authors:** P Zuercher, D Springe, A Putzu, D Grandgirard, S Leib, SM Jakob, J Takala, M Haenggi

**Affiliations:** University of Bern - Inselspital, Dept. of Intensive Care Medicine, Bern, Switzerland; University of Bern, Institute for Infectious Diseases, Neuroinfection Laboratory, Bern, Switzerland; Federal Office for Civil Protection, Biology Division, Spiez Laboratory, Spiez, Switzerland

## Introduction

The noble gas xenon exerts neuroprotective effects after various insults, but availability of xenon is limited. Helium and argon are readily available noble gases, but the results of studies using helium as neuroprotective measure are mixed, and the effects of argon are less well studied. In a recent cardiac arrest (CA) study, 70% argon (in O2) administered for 1 hour post CA improved outcome [1].

## Objectives

We examined the effect of prolonged administration of both gases as neuroprotective agents in a rat CA model.

## Methods

8-min asystolic cardiac arrest (intravenous KCl in esmolol) was induced in 48 male Wistar rats. 9 rats died during the operation/CA procedure, the remaining 39 were randomized into 3 groups: helium (h) (CA, 24 hours in a chamber with helium/O2 mixture of 50:50, starting 15 min after ROSC, n= 10), argon (a) (CA, 24 h in argon/O2 50:50, n=10) and control (c) (CA, 24 hours in O2/N2 50:50, n=11), 8 animals with surgery only served as sham animals (not randomized). 2 rats in the c group died after 7 h and 4.5 days. All surviving animals were euthanized at day 5.

The rats were assessed preoperatively and then daily until day 5 by a behavior score for rats, a Neuro-Deficit score and a Tape-Removal-Test. On day 0, 4 and 5 locomotor activity was recorded in an Open Field Test. The animals were then euthanized for harvest of brain for histology in the hippocampus cornus ammonis segment CA1, assessed with cresyl violet (CV) and Fluro-Jade (FJ) staining.

## Results

: CV staining demonstrated an absence of pyknotic cells in the sham group, compared to (h), (a) and (c), but the differences between the resuscitated rats were not significant (see table). This resulted in a non-significant decrease of the CA1 cell layer in the resuscitated animals. FJ staining demonstrated no cell damage in the sham group, but significant injury in the (h), (a) and (c) groups. Again, there was no significant difference between the resuscitated animals. No difference could be found in the neuropsychological and functional tests within the CA groups.

**Table 1:** Table 1

	helium/O2 n=10	argon/O2 n=10	air/O2 n=9	sham n=8	Sig./ Post hoc p<0.05
CV - pyknotic cells in CA1 [%]	49 [IQR 21 - 66]	52 [36- 86]	82 [50 - 92]	0 [0 - 1]	p<0.01 / sham vs all
CA1 cell layer (normalized: surface/length) [mm2/mm]	0.074 [0.060 - 0.084]	0.076 [0.059- 0.096]	0.067 [0.046- 0.089]	0.087 [0.081- 0.100]	p<0.01 / sham vs all
Fluoro-Jade [numbers of stained cells per mm]	148 [140 - 166]	154 [124 - 199]	145 [138 - 182]	0 [0 - 0]	p<0.01 / sham vs all

## Conclusions

In our 8 minutes cardiac arrest model with mild neurobehavioral damage neither the noble gas helium nor argon administered as a gas mix with 50% oxygen had a significant positive clinical effect. Post hypoxic-ischemic cell injury in the hippocampal CA1 segment did not differ between the helium, argon and control groups. Whether the neuroprotective effect of helium and argon is dose dependent remains open.
